# Perceptual confirmation bias and decision bias underlie adaptation to sequential regularities

**DOI:** 10.1167/jov.24.2.5

**Published:** 2024-02-21

**Authors:** Magdalena del Río, Floris P. de Lange, Matthias Fritsche, Jamie Ward

**Affiliations:** 1 School of Psychology, University of Sussex, Brighton, UK; 2 Donders Institute for Brain, Cognition and Behaviour, Radboud University, Nijmegen, the Netherlands; 3 Donders Institute for Brain, Cognition and Behaviour, Radboud University, Nijmegen, the Netherlands; 4 Department of Physiology, Anatomy & Genetics, University of Oxford, Oxford, UK; 5 School of Psychology, University of Sussex, Brighton, UK

**Keywords:** adaptation, choice history bias, perceptual decision-making, confirmation bias, autism spectrum disorder

## Abstract

Our perception does not depend exclusively on the immediate sensory input. It is also influenced by our internal predictions derived from prior observations and the temporal regularities of the environment, which can result in choice history biases. However, it is unclear how this flexible use of prior information to predict the future influences perceptual decisions. Prior information may bias decisions independently of the current sensory input, or it may modulate the weight of current sensory input based on its consistency with the expectation. To address this question, we used a visual decision-making task and manipulated the transitional probabilities between successive noisy grating stimuli. Using a reverse correlation analysis, we evaluated the contribution of stimulus-independent decision bias and stimulus-dependent sensitivity modulations to choice history biases. We found that both effects coexist, whereby there was increased bias to respond in line with the predicted orientation alongside modulations in perceptual sensitivity to favor perceptual information consistent with the prediction, akin to selective attention. Furthermore, at the individual differences level, we investigated the relationship between autistic-like traits and the adaptation of choice history biases to the sequential statistics of the environment. Over two studies, we found no convincing evidence of reduced adaptation to sequential regularities in individuals with high autistic-like traits. In sum, we present robust evidence for both perceptual confirmation bias and decision bias supporting adaptation to sequential regularities in the environment.

## Introduction

We often make sense of uncertain sensory information by exploiting the statistical regularities in our environment. For instance, we can leverage the fact that our world is reasonably stable over short timescales, such that the recent past is generally a good predictor of the present (van Bergen & Jehee, 2019). We can thus optimize perceptual decisions otherwise based on noisy input. Attractive choice history biases in perception, where the final perceptual decision is biased towards prior decisions, can be thought to arise as the result of a Bayesian integration process in a stable environment. Furthermore, it has been shown that observers can flexibly adapt choice history biases to the environment's statistics (Abrahamyan, Silva, Dakin, Carandini, & Gardner, 2016; Braun, Urai, & Donner, 2018; Glaze, Kable, & Gold, 2015; Urai, de Gee, Tsetsos, & Donner, 2019), thereby facilitating perceptual decisions in environments with different temporal regularities (e.g., where stimuli tend to repeat or alternate). However, the processes underlying this adaptation of choice history biases are largely unknown, and it is further unclear whether all individuals can adapt their history biases in the same way. In this study, we aimed to address these knowledge gaps.

Observers performed a standard perceptual decision-making task in which they indicated whether a target grating of varying contrast and embedded in visual noise was oriented clockwise or counterclockwise from vertical (45° or −45° from vertical). The environment statistics were manipulated by varying the transitional probabilities between successive stimulus orientations, which has been previously shown to induce an adaptation in choice history biases to approximate the sequential pattern (e.g., Braun et al., 2018). In an initial neutral environment, the stimulus orientation was equally likely to repeat or alternate. In a subsequent repeating environment, the stimulus orientation repeated with 80% probability.

The expectation induced by the learned sequential regularity may generate a bias signal, shifting decisions independent of the current incoming sensory information (e.g., de Lange, Rahnev, Donner, & Lau, 2013; Shadlen & Newsome, 2001). However, observers may also become more sensitive to the orientation information they predict to see, thereby overweighting sensory input consistent with their prediction, in line with a process akin to feature-based attention (Urai et al., 2019), leading to a perceptual confirmation bias (Talluri, Urai, Tsetsos, Usher, & Donner, 2018). Here, we evaluated both possibilities, which are not mutually exclusive, via reverse correlation analyses, capitalizing on arbitrary fluctuations in orientation signal in our target-absent noise stimuli (Wyart, Nobre, & Summerfield, 2012). This allowed us to quantify behavioral biases independent of the presented bottom-up sensory signal and the bias specifically for signals consistent with the predicted stimulus, which would point to a process akin to perceptual confirmation bias.

Furthermore, adaptation to a novel environment is likely to take place over time, yet previous psychophysical analyses assume the observer is stationary in time by averaging across multiple trials to estimate the observer's history biases. This limits the insights we can gain on the dynamics of learning statistical regularities. We therefore complemented the classical psychophysical analysis with a novel analysis using PsyTrack, a recently developed framework to estimate the psychometric parameters, including choice history biases, at the resolution of a single trial (Roy, Bak, Akrami, Brody, & Pillow, 2021).

In addition, we evaluated individual differences in adaptation to sequential regularities. Lieder et al. (2019) found that participants with autism spectrum disorders (ASD) exhibited a reduced influence of the recent stimulus history than neurotypical controls in a serial discrimination task, weighting recent information less strongly, which led the authors to propose that individuals with ASD are “slow adapters.” This is related to a broader literature which suggests impairments in the integration of immediate sensory evidence and long-term statistics (Brock, 2012; Lawson, Rees, & Friston, 2014; Pellicano & Burr, 2012; Sinha et al., 2014; Van de Cruys et al., 2014). Altered perceptual decision-making in ASD, with reduced reliance on prior choices, may thus stem from a failure to learn and exploit statistical regularities in the environment. Following this hypothesis, we predicted that the magnitude of choice history biases and in particular their adaptation to new sequential regularities would correlate negatively with autistic-like traits, as measured by the Autism Spectrum Quotient (AQ; Baron-Cohen, Wheelwright, Skinner, Martin, & Clubley, 2001), and the Glasgow Sensory Questionnaire (GSQ; Robertson & Simmons, 2013), a scale targeting the sensory issues characteristic of ASD, which are not captured by the AQ.

We find that both stimulus-independent bias and stimulus-dependent sensitivity modulations to choice history biases coexist, whereby there is increased bias to respond in line with the predicted orientation alongside modulations in perceptual sensitivity to favor information consistent with the prediction. At the individual differences level, we do not find convincing evidence of reduced choice history bias adaptation related to autistic-like traits. An initial sample of the ends of the AQ distribution in the general population suggested a potential effect in participants with particularly high scores, yet we did not replicate the effect in a sample of high-AQ participants who additionally self-reported receiving a diagnosis. Together, the study sheds light on how observers exploit sequential regularities to facilitate perceptual decision-making and suggests that the adaptation of choice history biases is not affected by autistic-like traits.

## Methods

Two online studies were conducted as pre-registered at https://osf.io/udsyp and https://osf.io/d8kgq respectively. Any deviations are reported and justified below.

### Participants

#### Study 1

Participants were recruited from the general population via Prolific in two stages. Stage 1 consisted of a screening survey comprising the AQ (Baron-Cohen et al., 2001) and the GSQ (Robertson & Simmons, 2013) and served to select individuals with high and low AQ eligible to participate in the second stage. In stage 2, this subset of participants completed the perceptual decision-making task.

**Figure 1.**
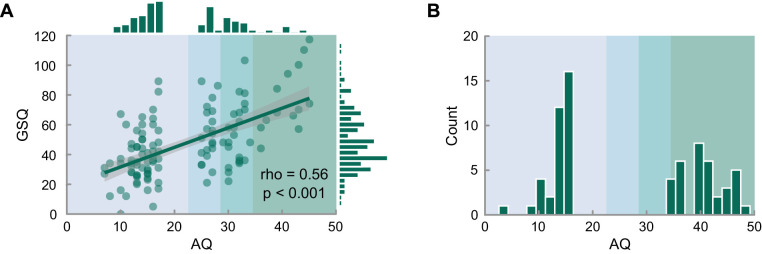
(**A**) Distribution of the total AQ and GSQ scores and their correlation in our final sample of 120 participants in study 1 and (**B**) distribution of the total AQ our final sample of 71 participants in study 2. Shades of green in the background indicate the categorical classification of AQ scores as neurotypical (AQ < 23, n = 63), broader autism phenotype (22 < AQ < 29, n = 21), medium autism phenotype AQ (28 < AQ < 35, n = 23), and narrow autism phenotype (AQ > 34, n = 13).

From our pool of 500 participants, who completed stage 1, we preferentially invited individuals with highest and lowest AQ scores to complete the second stage, thus creating two groups evenly distributed across high AQ scores (AQ > 24) and low AQ scores (AQ < 18). This deviated from the pre-registered approach of inviting all individuals with an AQ score above a prespecified cut-off and randomly selecting low-AQ participants due to the unexpected overrepresentation of high-AQ individuals in our stage 1 sample. Fifty-seven high-AQ participants (22 female, 33 male, 2 undisclosed, aged 21–69 [*M*_age_ = 40.1, *SD*_age_ = 12.4, *M*_AQ_ = 31.8, *SD*_AQ_ = 5.9]) and 63 low-AQ participants (38 female, 25 male, aged 20–72 [*M*_age_ = 43.4, *SD*_age_ = 11.3, *M*_AQ_ = 13.7, *SD*_AQ_ = 2.6]) completed the perceptual decision-making task (see Figure 1A).

The sample size was determined by means of a power analysis as well as practical considerations. The expected effect size was estimated based on previous studies investigating bias magnitude in various tasks either for two clinical groups, as in (Lieder et al., 2019; Cohen's *d* of 0.49), or according to variability in autistic-like traits in the general population, as in (Karvelis, Seitz, Lawrie, & Seriès, 2018; Cohen's *d* of 0.355) and (Lowe et al., 2018; Cohen's *d* of 0.559). Taking the mean Cohen's *d* of 0.468 as our expected effect size, the projected sample size estimated using G*Power for a one-tailed correlation with an *α* = 0.05, power = 0.8 was 115.

To follow up on the observed pattern in the data, we conducted a categorical analysis, where participants were further subdivided into four categories according to their AQ score, following the definitions of neurotypical, as well as broader, medium, and narrow autism phenotype (BAP, MAP and NAP respectively) proposed by Wheelwright, Auyeung, Allison, & Baron-Cohen (2010). Cutoff values for the classification of our sample were determined using the distribution of AQ scores for this large-scale study consisting of 1582 ASD families and 666 control families. AQ scores 1 SD above the mean AQ (22 < AQ < 29, n = 21) were classified as BAP, AQ scores 2 *SD*s above the mean AQ (28 < AQ < 35, n = 23), as MAP and AQ scores 3 SDs above the mean AQ (AQ > 34, n = 13), as NAP. Any AQ score below 1 *SD* above the mean AQ was classified as neurotypical (AQ < 23, n = 63). Because this analysis was not planned and most likely underpowered, we conducted a replication.

#### Study 2

Participants were recruited following the same procedure, whereby we exclusively invited NT (AQ < 17) and NAP (AQ > 34) individuals to participate in the second stage of the study and omitted the GSQ. We additionally prescreened participants, such that they were only eligible to participate in the study if they report having received a diagnosis of ASD either as a child or as an adult (NAP group) or having not received a diagnosis of ASD either as a child or as an adult (NT group), to maximize the chances of recruiting participants from the extreme ends of the distribution. Thirty-five high-AQ participants (11 female, 20 male, four undisclosed, aged 21–61 [*M*_age_ = 36.71, *SD*_age_ = 11.41, *M*_AQ_ = 13.69, *SD*_AQ_ = 2.53]) and 36 low-AQ participants (13 female, 23 male, aged 20–70 [*M*_age_ = 40.61, *SD*_age_ = 13.80, *M*_AQ_ = 40.63, *SD*_AQ_ = 3.83]) completed the perceptual decision-making task (see Figure 1B).

The sample size was determined by means of a power analysis based on study 1. Taking the smallest effect size comparing history bias adaptation for low-AQ versus NAP participants (Cohen's *d* of 0.606) as our expected effect size, the projected sample size estimated using G*Power for a one-tailed t test with an *α* = 0.05, power = 0.8 was 35 participants per group.

Participants were compensated at a rate of £7.5/hour. Participants reported normal or corrected-to-normal vision, and were additionally required to report fluency in English, a minimum of 400 previous submissions and a 90% approval rate on Prolific. The study was approved by the local ethics committee of the University of Sussex (reference number ER/MD517/10). All participants gave written informed consent.

### Stimuli and procedure

**Figure 2.**
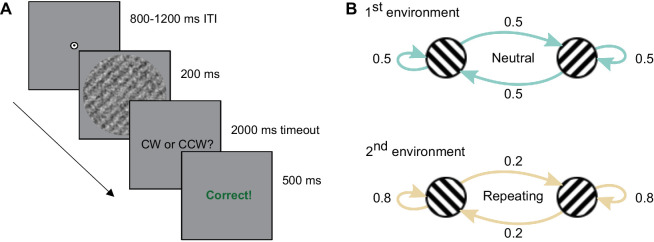
(**A**) Example trial: participants report the orientation of a target grating of varying contrast and embedded in noise which is tilted clockwise or counterclockwise (45° or −45°) from vertical (0°). Trial-wise and block-wise feedback was provided. (**B**) The transitional probability of stimulus orientations was manipulated to create two environments: an initial neutral environment (repetition probability of 0.5) and a subsequent repeating environment (repetition probability of 0.8).

Participants completed a standard two-alternative forced choice orientation discrimination task with trial-wise feedback in two environments (see Figure 2). In the initial neutral environment, the orientation of the stimulus (a grating embedded in white noise) was determined independently across trials and equally likely to be clockwise or counterclockwise, while in the repeating environment, the orientation of the stimulus was more likely to repeat across successive trials.

The experiment was implemented using PsychoPy and hosted on Pavlovia. Participants were first instructed to complete a card-matching task freely available on Pavlovia (https://pavlovia.org/Wake/screenscale) to estimate the monitors' logical pixel density and adjust the size of our stimuli accordingly. Next, they received detailed instructions including a demonstration trial, 3 slowed-down practice trials, and a practice block of 80 trials in the neutral environment. The main task consisted of 12 blocks of 100 trials each: Six blocks in the neutral environment, where the repetition probability of the stimulus orientation was 0.5, followed by six blocks in the repeating condition, where the repetition probability of the stimulus orientation was 0.8. The order of the environments was held constant across participants to avoid carryover effects of biases acquired in the repeating environment to the neutral environment.

Participants were instructed to focus on a central fixation cross throughout the trial and indicate if the grating was tilted clockwise or counterclockwise from vertical. Each trial consisted of a fixation period jittered between 800 and 1200 ms, a 200-ms stimulus presentation, and a response period of up to 2000 ms, during which participants indicated if the stimulus was tilted clockwise or counterclockwise by pressing the “m” or “x” key on the keyboard respectively. Feedback (the word “Correct” or “Incorrect” in red or green, respectively) was displayed after each trial for 500 ms. As additional feedback, participants were informed of their block-wise accuracy after each completed block. Missed trials triggered a warning message indicating that no response had been recorded and reminding participants to keep responding. An additional warning was presented after every 20 missed trials. Participants had the opportunity to take breaks after the demonstration trials, after the practice block and after each experimental block (the latter lasting a maximum of five minutes).

The following exclusion criteria were checked in real time, such that the experiment terminated automatically if they were not fulfilled:

1. Minimum performance of 65% accuracy in practice trials and in the main task (the latter was recomputed on each trial, after the first 200 trials had been recorded)
2. Maximum of 60 missed responses (5% of all 1200 trials)

In addition, we ensured the following criterion was met post-hoc:

3. Minimum performance of 65% accuracy in each of the two environments (blocks 1–6 and 7–12).

Images and condition matrices were generated in MATLAB using custom scripts. Gratings had a radius of approximately 5° of visual angle on a 40.8 cm wide monitor with a resolution of 1366 × 768 px at a viewing distance of 0.6 m. These were scaled based on participants’ logical pixel density derived from the card-matching task and the known monitor specifications of one of the experimenters to present a stimulus of approximately 10.6 cm in radius to all participants. Assuming a viewing distance of 0.6 m, gratings had a constant spatial frequency of 1 c/° , a random phase value between 0 and 1, and were embedded in noise with contrast of 0.3, smoothed with a Gaussian kernel with a SD of 0.08 visual degrees. The Michelson contrast of the target grating comprised six levels (0, 0.02, 0.04, 0.06, 0.12, and 0.18) and was fully randomized within each environment, ensuring equal numbers of presentations (except for the first 12 trials in the practice block, which were presented at full contrast for instruction purposes). In trials with 0%-contrast targets, the correct response was determined randomly in the neutral environment, whereas in the repeating environment it followed the sequential regularity and thus depended stochastically on the previous stimulus. A combination of easier and more difficult trials is needed to both establish the regularity of stimulus sequences and create perceptual uncertainty, which motivates the reliance on internal predictions during decision-making.

The serial-order counterbalanced orientation sequence in the neutral environment was determined using a specialized function in MATLAB which ensures that every condition is preceded equally often by every other condition (Brooks, 2012). The orientation sequence in the repeating environment was generated by sequentially sampling from a binomial distribution with a stimulus repetition probability of 0.8. This process was iterated over until the empirically determined repetition probability was within ±0.02 of the target repetition probability of 0.8.

### Probabilistic choice model

The goal of this analysis was to quantify each participant's history biases per environment, and thus confirm the presence of history bias adaptation to sequential regularities, prior to characterizing it further. We estimated each participant's probability of responding clockwise in a given trial of the neutral and repeating environment separately using an established multiple logistic regression model with lasso regularization (Fründ, Wichmann, & Macke, 2014). The model included regression coefficients for the current stimulus at trial t (target signal intensity, here the signed Michelson contrast of the grating), the history-independent bias to respond clockwise, the lapse rates (the rate of clockwise and counterclockwise responses independent of stimulus intensity), and the stimuli and choices in the previous seven trials. The choice of temporal horizon (number of previous stimulus and response regressors) was motivated by the fact that the autocorrelation of the repeating sequence approaches zero for lags above 7, and is in line with the temporal horizon used in previous studies (e.g., Braun et al., 2018). The regularization parameter λ was set to 0.001. To ensure that with this regularization we could accurately recover history biases in the repeating environment, in which current and previous stimuli were correlated, we ran a simulation procedure. We first estimated psychometric parameters (intercept, slope, lapse rates, and history biases) for each participant in the uncorrelated, neutral environment. Using these parameters, we then simulated 100 sets of responses to the stimulus sequences of the repeating environment of each participant. Subsequently, we repeated the lasso regression on these simulated responses. The simulation confirmed that we could accurately recover the simulated history biases in the repeating environment (see Supplementary Figure S1).

The analysis focused on the previous correct choice regressor in each environment and the adaptation of the previous correct choice regressor after transitioning from the neutral to the repeating environment (previous correct choice regressor in the repeating environment minus previous correct choice regressor in the neutral environment). We had predicted that the adaptation effect would be more pronounced for the stimulus regressor as opposed to the choice regressor, as the trial-wise feedback provided participants with the ground-truth orientation information. We therefore expected participants to use this to predict the upcoming stimulus instead of their previous, potentially incorrect choice. However, we observed that the full effect is better captured by summing previous stimulus and previous choice weights, which is mathematically equivalent to the previous correct choice weight. We consider this to be the more appropriate measure of history effects in our data, yet this was not pre-registered and we thus report the associations with the previous stimulus and choice regressors in full. As a control analysis, we also report the correlations of autistic-like traits with the current stimulus regressor, the intercept and the lapse rates, as well as the mean accuracy and reaction time.

### Time-resolved probabilistic choice model

Sequential regularities need to be learned by experience, such that we would expect adaptation to the repeating environment to not be instantaneous, but rather to develop over time. We therefore aimed to quantify time-resolved history biases per environment. We estimated the development of each observer's choice history biases over time in the neutral and repeating environment separately using PsyTrack (Roy et al., 2021). The model of the probability to respond clockwise included regression coefficients for the current stimulus at trial t (target signal intensity, here the z-scored Michelson contrast of the grating), the history-independent bias to respond clockwise, and the previous response and previous stimulus regressors in the previous seven trials, thus matching the temporal horizon of the time-unresolved analysis. Regressors are estimated on every trial, with each of these evolving according to a Gaussian random walk. The standard deviation σ characterizing the average rate of change of the weight across trials was initialized at the default value of 2^−^^5^, and the variability on the first trial was set at the larger value of 2^5^ to allow the data to determine the starting point.

Given the clear linearity of the curves, the learning rate metric was based on the intercept and slope of the linear fit to the individual history weight trajectory in the repeating environment. We had preregistered a Bayesian model comparison to determine the optimal function describing the change in history regressors over time against each other (specifically, a null model with no difference in weights across trials, a switch-point model where there is an abrupt change in weights, a linear model and a quadratic model). However, this step was redundant in light of the results.

### Reverse correlation analysis

To investigate how choice history affected perceptual decisions, we utilized the noise-driven fluctuations in the orientation signal of the 0%-contrast stimuli in a reverse-correlation analysis. We estimated the probability of an observer responding clockwise, together with their stimulus-independent bias and stimulus-dependent sensitivity to orientation information fluctuations separately for trials preceded by a counterclockwise and a clockwise stimulus in each environment. This allowed us to compare effects of choice history on both stimulus-independent decision bias and stimulus-dependent sensitivity.

The amount of signal energy present in each noise stimulus was quantified through convolution of the image with a pool of Gabor filters with tuning properties comprising all combinations of orientations between −89° to 90° in steps of 1° and spatial frequencies ranging from 0.6 to 2.5 c/° in steps of 0.1 c/° ( i.e., the target frequency of 1 ± 0.4 c/°, using custom MATLAB scripts). To test the effect of the previous stimulus (and the prediction derived thereof) on the current trial, the data was then split according to the environment (neutral or repeating) and the orientation of the previous stimulus (clockwise or counterclockwise). Each spatial-frequency bin within each dataset was fit with a logistic regression model using custom scripts in *R*, to estimate the probability of the observer responding clockwise as *P*(clockwise) = Φ (*β*0 + *β*1 · *Z*[E(S)]). Here Φ is the logistic function, *β*0 is the intercept (bias), *β1* scales the relationship between the orientation energy and the response (sensitivity), and *Z*[E(S)] is the orientation energy of a given orientation and spatial frequency combination, z-scored per orientation, spatial frequency, participant, previous stimulus orientation and environment. We subsequently removed outliers, defined as values outside 1.5 times the interquartile range, calculated in turn by pooling estimates for all participants, orientation and spatial frequency combinations contingent on the previous stimulus (clockwise or counterclockwise) and the environment (neutral or repeating). Summary statistics were created by averaging the sensitivity regressors (*β*1), corresponding to clockwise orientations (89° to 1°) and counterclockwise orientations (−89°, −1°) and all spatial frequencies (0.6 to 1.4 c/°). The bias regressors (*β*0) were averaged across all orientations (−89°, 89°) and all spatial frequencies (0.6 to 1.4 c/°), because the term is stimulus-independent. This resulted in an average bias *β*0 per previous stimulus orientation (clockwise or counterclockwise), and per environment (neutral or repeating), and an average sensitivity *β*1 per orientation energy in the noise (clockwise or counterclockwise), per previous stimulus orientation (clockwise or counterclockwise), and per environment (neutral or repeating) for each participant.

Deviating from our preregistered analysis, we focused our reverse-correlation analysis on 0% contrast trials, in which observers’ responses were purely driven by internal biases and fluctuations in orientation energy of the white noise stimuli. This eliminated the need to account for the influence of the grating signals on the observers’ responses, allowing for a more straightforward assessment of our main hypotheses regarding the sensitivity to noise-driven fluctuations in orientation signals.

We had additionally preregistered an exploratory analysis investigating the specificity of the stimulus-dependent β1 regressor resolved by orientation and spatial frequency by means of a cluster-based permutation test in the 2-dimensional space spanned by orientation and spatial frequency. This was omitted in light of the broad orientation tuning of the energy sensitivity profiles (see Figure 5).

### Statistical tests

The hypotheses relating to the behavioral effects of history biases were tested by means of paired t tests in the case of the bias term (previous stimulus orientation clockwise vs previous stimulus orientation counterclockwise separately in each environment), and a 2 × 2 repeated-measures analysis of variance in the case of the sensitivity term (previous stimulus orientation clockwise vs. previous stimulus orientation counterclockwise, and clockwise orientation energy vs. counterclockwise orientation energy, separately in each environment). In addition, the environment was included as a factor in a 2 × 2 or a 2 × 2 × 2 repeated-measures analysis of variance, for the bias term and the sensitivity term, respectively.

The relationship between the measures of choice history biases across time-resolved, time-unresolved, and reverse-correlation analyses was assessed using Pearson's correlations. To compare the estimates of the time-resolved and -unresolved models, we averaged the one-back stimulus and response weights across trials for each environment and participant.

Individual differences throughout were assessed by means of Spearman's rank correlation between each of the measures and the AQ and GSQ scores. Group differences between the neurotypical and NAP samples were tested using Welch's *t* tests. Note that, because this analysis was not planned, we conducted a replication study specifically recruiting low-AQ and NAP participants, and using one-sided Welch's *t* tests.

Bayes factors were calculated using JASP with default parameters. Bayes factors for main and interaction effects are reported as *BF*_incl_ and are derived by comparing the model with the effect of interest vs the model without the effect of interest, all else being equal. Deidentified data and analysis code are available on OSF: https://doi.org/10.17605/OSF.IO/9K2TP.

## Results

### Observers adapt their history biases to the statistics of a repeating environment

**Figure 3.**
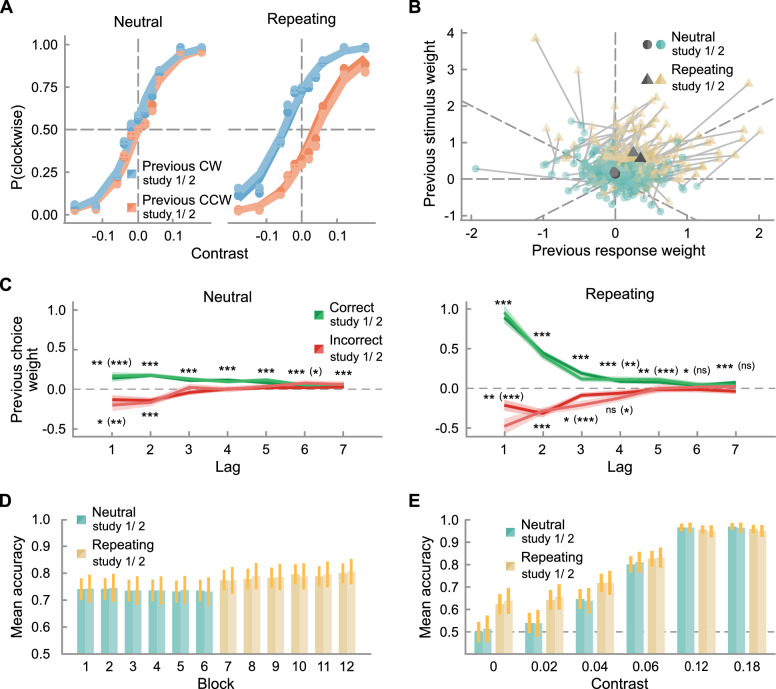
Adaptation of history biases to the environment statistics: (**A**) Group-average psychometric curves in the neutral and repeating environments, each conditioned on the stimulus orientation in the previous trial. Filled circles show the empirical data, lines the model prediction. (**B**) Effect of the one-back previous stimulus and previous response on the current response. Blue circles and yellow triangles indicate the weights for individual observers in neutral and repeating environments respectively, estimated with multiple logistic regression—each observer's estimates are connected by a gray line. The larger dark gray circle and triangle show the group-average weights in the neutral and repeating environments respectively for study 1 and study 2. (**C**) Previous correct and incorrect choice weights as a function of lag in the neutral and the repeating environment for study 1 and study 2. Shaded areas depict the standard error of the mean and asterisks the level of significance (*p < 0.05, **p < 0.01, ***p < 0.001). Asterisks in parentheses refer to study 2, where the level of significance does not coincide. (**D**) Mean accuracy per block for study 1 and study 2, and (**E**) per contrast and environment (blocks 1–6 in the neutral environment in blue, blocks 7–12 in the repeating environment in brown, dashed line shows chance performance) for study 1 and study 2. Error bars depict the standard error of the mean.

Participants successfully adapted their history biases to the repeating environment statistics. This is evidenced by the clear shift in the psychometric curves conditioned on the stimulus orientation in the previous trial in the repeating environment (see Figure 3A). Our multiple logistic regression analysis confirmed that participants increased their bias towards the stimulus orientation of the previous trial in the repeating versus the neutral environment (Figure 3B, study 1: *t*(119) = 9.37, *p* < 0.001, *BF*_10_ = 1.09 × 10^13^; study 2: *t*(70) = 8.33, *p* < 0.001, *BF*_10_ = 1.95 × 10^9^). Along with this adaptation in the previous stimulus weight, participants also increased their bias to repeat their previous response (Figure 3B, study 1: *t*(119) = 9.61, *p* < 0.001, BF_10_ = 3.71 × 10^13^; study 2: *t*(70) = 7.34, *p* < 0.001, *BF*_10_ = 3.42 × 10^7^). The simultaneous increase of previous stimulus- and choice-weight reflects the participants’ tendency to repeat their choices after correct trials, and a much weaker tendency to switch choices after incorrect trials (Figure 3B). We therefore focused our subsequent analyses on history biases following correct trials, computed by summing previous stimulus and choice weights. Both previous correct and incorrect weights decay with increasing temporal distance, with *t* tests against zero remaining significant for a lag of up to 7 (study 2: 5) trials for the previous correct choice weight and up to 3 (study 2: 4) trials for the previous incorrect choice weight (all *p* < 0.05) in the repeating environment (see Figure 3C). History bias adaptation to the repeating sequence provided an advantage in task performance, driven by an improvement in accuracy in low-contrast trials, defined here as contrasts below 0.06% (see Figures 3D and 3E). Indeed, the magnitude of the adaptation of the previous correct choice weight correlated with accuracy in low-contrast trials in the repeating environment (study 1: *ρ*_s_ = 0.49, *p* < 0.001, *r* = 0.48 [0.32, 0.60], *BF*_10_ = 350428.07; study 2: *ρ*_s_ = 0.65, *p* < 0.001, *r* = 0.67 [0.51, 0.78], *BF*_10_ = 9.99 × 10^7^). Importantly, it was not possible to predict the rewarded response in 0%-contrast trials in the neutral environment, because these were determined randomly, leading to obligatory chance-level performance of 50% accuracy. Conversely, in the repeating environment, exploiting the transitional structure, which also applied to 0%-contrast trials, allowed participants to increase their accuracy above 50% (Figure 3E). Overall, our results show that participant successfully adapted their choice history biases to the repeating temporal regularity, thereby improving their perceptual decisions.

### Adaptation to repeating temporal regularity is supported by concurrent perceptual confirmation bias and decision bias

Although observers successfully adapted their history biases to the sequential regularities of the repeating environment, it is unclear how these history biases and their adaptation manifest. We had hypothesized that observers could exploit their perceptual history in two, not mutually exclusive ways. First, observers could offset the accumulated sensory evidence in favor of the predicted stimulus orientation independently of the presented signal (stimulus-independent bias). Second, observers could preferentially accumulate evidence that is consistent with the predicted stimulus orientation (stimulus-dependent sensitivity), leading to a perceptual confirmation bias.

**Figure 4.**
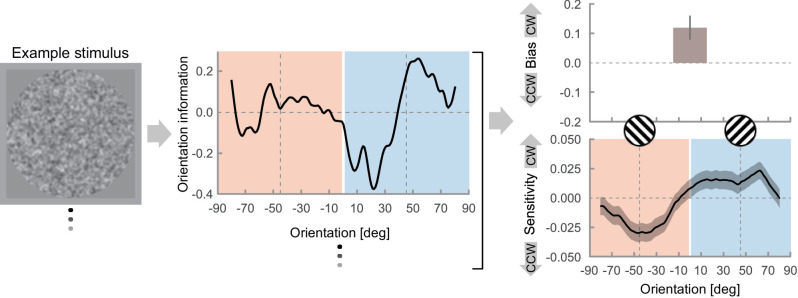
Schematic of the reverse-correlation analysis: Noise-driven fluctuations in the orientation information in 0%-contrast trials (left) are quantified in the orientation energy profile of the image (center), computed by convolving the image with a pool of Gabor filters tuned to all combinations of orientation between −89° to 90° in steps of 1° and spatial frequency between 0.6 to 1.4 c/° in steps of 0.1 c/° (i.e., the target frequency of 1 ± 0.4 c/°). Using the orientation energy profiles of all 0%-contrast target stimuli to predict participants’ probability to respond clockwise vs counterclockwise allowed us to estimate a stimulus-independent bias and their sensitivity to the noise-driven orientation energy (top and bottom right, respectively ; data generated by averaging across all trials in the neutral environment and all participants). History bias adaptation to the environment statistics can be assessed by conditioning this model on the target orientation in the previous trial (i.e., by splitting the data according to previous stimulus orientation and environment). Shaded areas in the background indicate the categorical distinction between orientation information in the noise which is clockwise from vertical or 0° (positive values, in blue) and counterclockwise from vertical (negative values, in red), yet note that the sensitivity was estimated continuously in steps of 1°.

These effects (stimulus-independent bias and stimulus-dependent sensitivity to predicted and unpredicted orientations) were estimated based on responses to the 0%-contrast trials (i.e., trials that did not contain a target grating and consisted solely of random fluctuations in orientation information in the noise stimulus). The orientation information in the noise stimulus was quantified by convolving the image with a pool of Gabor filters tuned to a range of spatial frequency and orientation combinations. Based on these orientation energy profiles derived from the noise stimuli, we predicted the probability of an observer responding clockwise on a given trial using logistic regression. This allowed us to quantify both the sensitivity to noise-driven orientation energy, as well as overall biases to respond clockwise or counterclockwise. By additionally conditioning our data on the previous stimulus orientation (clockwise vs. counterclockwise), we were able to compare history effects on bias and sensitivity (see the method schematic in Figure 4).

**Figure 5.**
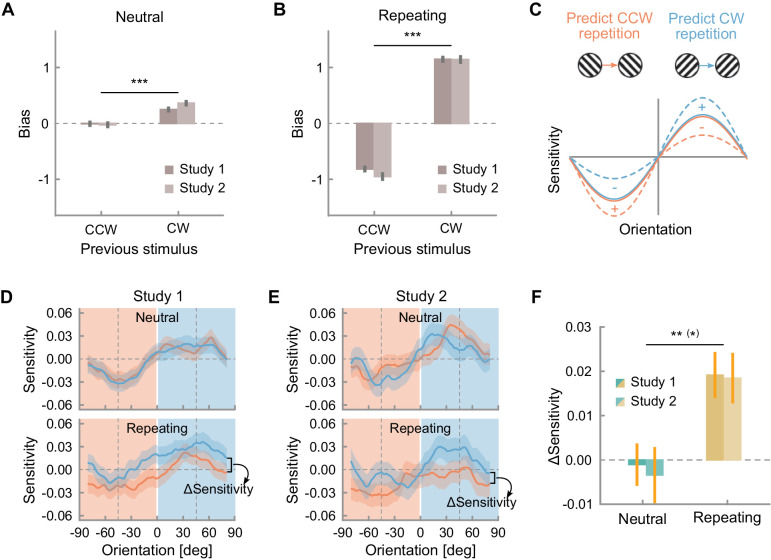
Stimulus-independent bias in (**A**) the neutral and (**B**) the repeating environment, conditioned on the previous stimulus orientation (clockwise or counterclockwise). (**C**) Schematic of the expected modulation of stimulus-dependent sensitivity in line with predicted orientation information after adaptation to a repeating environment. Stimulus-dependent sensitivity to orientation information in the noise in the neutral and the repeating environment in (**D**) study 1 and (**E**) study 2, conditioned on the previous stimulus orientation. Shaded areas in the background indicate the categorical distinction between orientation information in the noise which is clockwise from vertical or 0° (positive values, in blue) and counterclockwise from vertical (negative values, in red), yet note that the sensitivity was estimated continuously in steps of 1°. (**F**) History effect on stimulus-dependent sensitivity (sensitivity to clockwise orientation information in the noise for trials where the previous stimulus was clockwise minus sensitivity to clockwise orientation information in the noise for trials where the previous stimulus was counterclockwise) in the neutral and repeating environments. Error bars and shaded areas depict the standard error of the mean and asterisks the level of significance (*p < 0.05, **p < 0.01, ***p < 0.001). Asterisks in parentheses refer to study 2, where the level of significance does not coincide.

Whereas we had hypothesized that observers would become either more biased or more sensitive to the predicted orientation, we found evidence of both effects taking place concurrently. Observers had a stimulus-independent repetition bias already in the neutral environment (study 1: *t*(118) = 3.43, *p* < 0.001, *BF*_10_ = 24.73; study 2: *t*(69) = 4.90, *p* < 0.001, *BF*_10_ = 2830.78; see Figure 5A), which was exacerbated in the repeating environment (study 1: *t*(118) = 20.30, *p* < 0.001, *BF*_10_ = 8.82 × 10^36^, study 2: *t*(67) = 15.67, *p* < 0.001, *BF*_10_ = 9.25 × 10^20^; interaction between previous stimulus and environment— study 1: *F*(1, 355) = 248.83, *p* < 0.001, *BF*_incl_ = 4.53 × 10^58^; study 2: *F*(1, 206) = 161.41, *p* < 0.001, *BF*_incl_ = 9.50 × 10^33^; see Figure 5B). Nonetheless, observers were sensitive to the fluctuations in the noise-driven orientation energy independently of the presence of sequential regularities (neutral environment— study 1: *F*(1, 357) = 61.48, *p* < 0.001; *BF*_incl_ = 2.92 × 10^13^; study 2: *F*(1, 210) = 49.88, *p* < 0.001; *BF*_incl_ = 2.58 × 10^10^; repeating environment: *F*(1, 357) = 38.51, *p* < 0.001, *BF*_incl_ = 9.64 × 10^7^; study 2: *F*(1, 210) = 17.43, *p* < 0.001; *BF*_incl_ = 903.17). Crucially though, only in the repeating environment is sensitivity modulated by the previous stimulus (neutral environment— study 1: *F*(1, 357) = 0.08, *p* = 0.77, *BF*_incl_ = 0.18; study 2: *F*(1, 210) = 0.55, *p* = 0.46; *BF*_incl_ = 0.28; repeating environment: *F*(1, 357) = 22.93, *p* < 0.001, *BF*_incl_ = 68.26; study 2: *F*(1, 210) = 16.68, *p* < 0.001; *BF*_incl_ = 18.46; interaction between previous stimulus and environment— study 1: *F*(1, 833) = 13.16, *p* < 0.001, *BF*_incl_ = 6.55; study 2: *F*(1, 490) = 10.39, *p* = 0.001; *BF*_incl_ = 4.09; Figure 5D– F). In other words, in the presence of sequential regularities, the information in line with the predicted orientation is amplified, whereas information against the predicted orientation is suppressed. Neither of these processes (amplification or suppression, shown as + and −, respectively, in Figure 5C) appears to conclusively dominate over the other, because a paired *t* test comparing sensitivity adaptation for orientations consistent versus inconsistent with the prediction is not robustly significant (study 1: *t*(119) = −0.90, *p* = 0.37, *BF*_10_ = 0.15; study 2: *t*(1, 70) = −2.58, *p* = 0.012; *BF*_10_ = 2.77). In summary, observers exploit the sequential statistics by adjusting both their stimulus-independent bias and their sensitivity to predicted and unpredicted sensory information.

**Figure 6.**
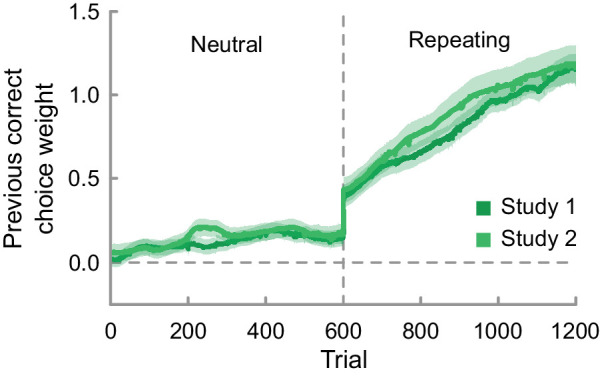
Trajectory of the previous correct choice bias across time in an initial neutral and subsequent repeating environment for all participants. Shaded areas depict the standard error of the mean.

**Figure 7.**
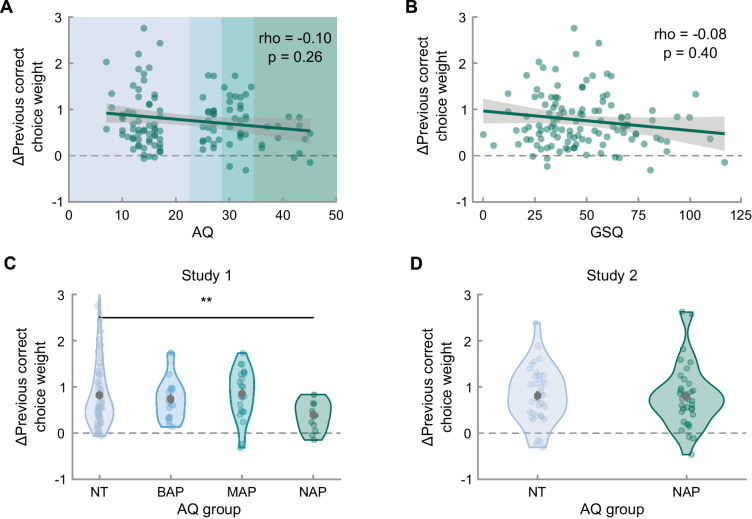
Individual differences in adaptation of the previous correct choice bias to the environment statistics related to autistic-like traits based on (**A**) the continuous distribution of AQ scores in study 1, (**B**) the continuous distribution of GSQ scores in study 1, (**C**) a categorical classification based on the AQ as neurotypical (NT; AQ < 23, n = 63), broader autism phenotype (BAP; 22 < AQ < 29, n = 21), medium autism phenotype AQ (MAP; 28<AQ < 35, n = 23), and narrow autism phenotype (NAP; AQ > 34, n = 13) in study 1, and (**D**) the categorical classification based on the AQ as NT (AQ < 17, n = 36) and NAP (AQ > 34, n = 35) as well as self-reported diagnosis, in study 2. Shades of blue in the background in A indicate the categorical classification of AQ scores. Error bars depict the standard error of the mean and asterisks the level of significance (*p < 0.05, **p < 0.01, ***p < 0.001).

### Choice history bias adaptation develops over time

The time-resolved history bias model largely confirms the results of the time-unresolved model (time-unresolved and time-resolved estimates averaged across trials are in high agreement— study 1: previous correct neutral: *r* = 0.98, *p* < 0.001; previous correct repeating: *r* = 0.96, *p* < 0.001; study 2: previous correct neutral: *r* = 0.97, *p* < 0.001; previous correct repeating: *r* = 0.96, *p* < 0.001) and extends the findings by characterizing the dynamics of the history bias development (see Figure 6). Averaging across all participants, the previous correct choice bias remained relatively constant and close to zero over the course of the six blocks in the neutral environment. Switching to the repeating environment leads to an initial sharp increase followed by a more gradual positive progression of the trajectory. As the development of the weights is on average linear, the learning rate can be indexed by the intercept and the slope of a linear fit of the previous correct choice weight trajectory in the repeating environment. Briefly, the time-resolved analysis reveals rapid as well as gradual adaptation of choice history weights over the course of exposure to a new sequential regularity.

### Similar adaptation for low- and high-AQ participants

Our previous analyses characterize the effects of choice history adaptation and additionally reveal large individual variability. We had hypothesized that this variability would be related to autistic-like traits, such that individuals with higher levels of autistic-like traits would show a reduced ability to learn and exploit sequential regularities, which would manifest as a reduced adaptation of history biases after transitioning to a repeating environment. In study 1, we found no correlation with the GSQ, a scale which specifically targets sensory issues characteristic of ASD, (*ρ*_s_ = −0.08, *p* = 0.26, *r* = −0.15 [−0.32, 0.03], *BF*_10_ = 0.43, see Figure 7B), and a trend for reduced adaptation of the previous correct choice weights in individuals with higher AQ scores (*ρ*_s_ = −0.10, *p* = 0.26, *r* = −0.16 [−0.33, 0.02], *BF*_10_ = 0.54, see Figure 7A), which appeared driven by the end of the spectrum. Following up on the pattern in the data, we divided the sample into subgroups defined in the literature based on the distribution of the AQ in the general population (Wheelwright et al., 2010), namely broader, medium and narrow autism phenotypes (BAP, MAP, and NAP). Using this categorical approach, we found a significant reduction in history bias adaptation in the NAP compared to the neurotypical group (*t*(41.86) = −3.37, *p* = 0.002, *BF*_10_ = 1.69, see Figure 7C). However, we did not replicate this group difference in study 2, in which we specifically recruited low-AQ and NAP participants who self-reported having received a diagnosis (*t*(67.78) = −0.14, *p* = 0.45, *BF*_10_ = 0.25, see Figure 7D). In short, we did not find evidence for reduced adaptation of history weights across the AQ spectrum, even after targeted recruitment of a NAP group. The remaining analyses of individual differences likewise did not replicate and can be found in the supplementary materials.

## Discussion

Statistical regularities in the environment allow us to optimize our perceptual decisions, nudging our choices towards the more likely option. Here we used a reverse-correlation analysis to show that this flexible adaptation to the sequential structure in the environment is underpinned by both an increase in stimulus-independent bias to the predicted orientation and a shift in stimulus-dependent sensitivity to sensory information which amplifies the influence of predicted information and suppresses that of unpredicted information. In parallel, we found considerable individual differences in the degree of adaptation to new regularities in sequences of visual input, yet no convincing evidence for reduced adaptation in individuals with high levels of autistic-like traits.

The initial neutral environment provides a baseline measure of the observer's inherent choice history biases. In line with previous studies, we find substantial idiosyncratic variability in these inherent biases, such that, although repetition biases predominate, some participants have alternation biases (Abrahamyan et al., 2016; Urai et al., 2019). Although seemingly irrational, inherent history biases in response to fully randomized stimulus sequences have been documented extensively and are generally thought to reflect an individual's expectation of structure in the environment and consequent detection of spurious patterns (Glaze et al., 2015; Yu & Cohen, 2008). However, this idiosyncratic a priori expectation of the environment structure is modifiable, as observers (both humans and rodents) adjust their history biases when exposed to non-random sequential structure in the environment (Abrahamyan et al., 2016; Braun et al., 2018; Fritsche et. al, 2023; Hermoso-Mendizabal et al., 2020). Indeed, we successfully replicated this history bias adaptation effect in an online study: on average, participants had a higher tendency to repeat previous correct choices when they transitioned from a completely random stimulus sequence to a sequence in which stimuli were more likely to repeat. It is noteworthy that overall this suggests flexibility in adapting to statistical regularities, yet less so to their absence. Perhaps part of the reason is that random sequences do not provide a strong expectation violation— rather there is essentially absence of evidence for any statistical regularity and most likely occasional spurious patterns that align with inherent biases by chance. In line with this, theoretical models for the dynamics of hidden biases exhibit surprisingly high probabilities of misidentifying random sequences as biased (Bialek, 2005). Human observers may therefore perceive little evidence for changing their intrinsic choice history biases when responding to random sequences. Moreover, Abrahamyan et al. (2016) show that the adaptation of choice history biases is more pronounced if the presented regularity is in line with the observers’ inherent biases. This may extend to adapting to spurious local sequential regularities in random sequences congruent with existing biases, thereby maintaining such biases in the face of randomness.

The underpinnings of such adaptations of choice history biases to temporal regularities remain unclear. The expectations induced by trial history could bias perceptual decisions by means of a shift in decision criterion independent of the presented sensory information. Observers could alternatively become more sensitive to predicted information by means of a gain modulation mechanism, akin to selective attention and therefore contingent on the information which is actually being presented (Urai et al., 2019). These possibilities are not mutually exclusive, and indeed here we find evidence of a concurrent adaptation of stimulus-independent bias and stimulus-dependent sensitivity to predicted information, whereby the effect of the stimulus-independent bias predominates. The increase in sensitivity is consistent with the results of the reverse-correlation analysis performed by Wyart et al. (2012). Our analysis expands on these previous findings by (a) extending the effect to a manipulation of expectation based on a probabilistic sequential pattern as opposed to a cue association and (b) applying a discrimination task instead of a detection task, which allows for the detection of changes in sensitivity to unpredicted as well as predicted information. Likewise, the results dovetail with alternative analysis strategies, which also find evidence for sensitivity being modulated by choice and stimulus history. Murai and Whitney (2021) argue that the previously seen stimulus shapes the perceptual template using an image classification approach. Urai et al. (2019) fit drift-diffusion models on reaction times and show evidence that accumulation biases as opposed to starting point biases explain individual differences in choice repetition behavior. Importantly, however, these studies investigated “default” history biases in response to randomized stimulus sequences, whereas we additionally manipulated temporal input regularities and thus focused on adaptive history biases.

More broadly, the increase in sensitivity for expected stimuli is consistent with a Bayesian theory of perception, where perceptual content is biased toward the expected, as opposed to a cancellation theory, where perception is biased towards the unexpected because it is more informative, and would therefore predict higher sensitivity for unexpected stimuli (Press, Kok, & Yon, 2020). Although the current studies were not designed to disentangle low- and high-level effects of perceptual choice history biases (Cicchini, Mikellidou, & Burr, 2017; Cicchini, Benedetto, & Burr, 2021; Fritsche, Mostert, & de Lange, 2017; Pascucci et al., 2019), overall the pattern of results for the stimulus-dependent sensitivity term resembles the classic description of selective attention, where the signal-to-noise ratio or tuning of the stimulus channels is modulated to strengthen the representation of the focus of attention (e.g., Martínez-Trujillo & Treue, 2004). This possibility is supported by Wyart et al. (2012), who conducted a simulation analysis to show that only biased baseline activity in signal-tuned neurons and not a shift in the decision criterion can produce sensitivity effects similar to those we observed in an environment with statistical regularities. Indeed, this mechanism has been suggested to underly serial dependence effects (Fischer & Whitney, 2014, also see Pascucci et al. (2023) and Manassi, Murai, & Whitney (2023) for relevant reviews), and potentially confirmation bias (Prat-Ortega & Rocha, 2018), a known widespread phenomenon in the domain of higher cognition and reasoning (Nickerson, 1998). The phenomenon has recently been highlighted in the perceptual domain, as observers have been shown to weight a second set of stimuli more strongly when it was consistent with their preliminary response than when it was inconsistent, a classic example of confirmation bias (Bronfman et al., 2015; Talluri et al., 2018). Here, in the context of the repeating environment, we found a similar effect taking place, in the absence of an explicit instruction to integrate across sensory inputs of successive trials. The specificity of the sensitivity modulation to the repeating environment might suggest a partial dissociation of the decision bias and sensitivity effects. However, it remains to be conclusively determined whether bias and sensitivity modulations are supported by different neural processes, as it is also possible that a single neural mechanism may result in distinct behavioral manifestations. To this point, the slight asymmetry and vertical shifts in Figure 5D may suggest a potential additional mechanism involving a shift in tuning channels around 0° depending on prior expectations. However, we consider it more likely that this asymmetry arose through measurement noise (e.g., spurious correlations between noise-driven energy of different orientations), as well as the computation of a moving average over an asymmetric change. Importantly, there was no evidence of a vertical shift in study 2, using the same experimental paradigm as study 1.

The dynamics of updates to the internal model of the environment statistics are not well characterized to date, though previous studies have shown a build-up of serial dependence over time (Barbosa & Compte, 2020). Our time-resolved analysis shows that choice history biases remain relatively constant and close to zero over the course of the blocks pertaining to the neutral environment. Switching to the repeating environment results in an initial sharp increase in choice history biases, followed by a more gradual positive progression of the trajectory until the end of the experiment, quantified respectively as the intercept and the slope of the linear fit. Because it is clearly impossible to adapt to a sequential pattern instantaneously, we interpret the differentiation between intercept and slope to reflect the distinction between a fast-acting adaptation process, presumably related to counting transitions over an immediately preceding trial window of a certain fixed length, and a slower continuous learning of the global environment statistics (here of a repeating sequential structure). However, we would caution against a strict mapping of these processes to the intercept and slope as estimated here, given that in our data intercept and slope are negatively correlated in all environments (neutral environment— study 1: *r* = −0.42, *p* < 0.001; study 2: *r* = −0.58, *p* < 0.001; repeating environment— study 1: *r* = −0.48, *p* < 0.001; study 2: *r* = −0.36, *p* = 0.002; adaptation— study 1: *r* = −0.59, *p* < 0.001; study 2: *r* = −0.59, *p* < 0.001). Although it is plausible that a trade-off between fast and slow learning processes is taking place, because observers who learn fast have less room for improvement and vice versa, the correlation in the neutral environment in particular suggests some limitations in the Psytrack algorithm in clearly disambiguating fast and slow learning processes as intercept and slope.

In this context, we note that two study design choices may affect the learning dynamics. First, we provided participants with access to the “ground truth” through explicit feedback, which most likely accelerated the learning process. Second, we chose a fixed-order experimental design, starting the session with the neutral environment as opposed to the repeating one, to obtain a baseline measure of history biases while avoiding crossover repetition bias effects in the neutral environment. Because our data and previous literature (Glaze et al., 2015; Yu & Cohen, 2008) suggest that observers do not tend to suppress history biases in response to fully randomized environments, it appears unlikely our design choice caused cross-over effects in the repeating environment. However, it is possible that the initial environment plays a role in setting the participants’ higher-level expectations of statistical regularities or their volatility and thus modulates the learning dynamics. For example, starting the experiment directly in a repeating environment with no prior exposure to a neutral one might potentially accelerate learning. These would be testable hypotheses for future studies.

A more nuanced understanding of choice history bias adaptation may be crucial to pinpointing the source(s) of individual differences. Despite the recent popularity of theories postulating an imbalance in the integration of prior and sensory information (e.g., Brock, 2012; Pellicano & Burr, 2012; Sinha et al., 2014), there is mixed evidence on choice history biases being atypical in ASD, with previous studies reporting decreased (Lieder et al., 2019), increased (Feigin, Shalom-Sperber, Zachor, & Zaidel, 2021) and unaltered choice history biases (Bosch, Fritsche, Utzerath, Buitelaar, & de Lange, 2022). Because a broader literature has suggested that altered perceptual processing may be due to atypical learning of the environment statistics (Karvelis et al., 2018; Lawson et al., 2014; Noel, Zhang, Stocker, & Angelaki, 2021), here we had hypothesized that the reliance on choice history might be particularly affected when adapting to a new sequential structure. In light of the null results and increasing body of mixed findings, we highlight two points of consideration for future research. First, it is unclear whether there exists a general capacity for statistical learning per se which would cut across cognitive domains, sensory modalities and contexts (Bogaerts, Siegelman, Christiansen, & Frost, 2022). Relatedly, it is unclear to what extent all choice history dependencies in perceptual decision-making paradigms are driven by the same underlying mechanisms. Although Urai et al. (2019) find consistent history-modulations of sensory evidence accumulation across different tasks and modalities, in broad agreement with the current results, there is also evidence for different visual features having different temporal serial dependence properties (Taubert, Alais, & Burr, 2016). Furthermore, alternative and not mutually exclusive mechanisms, such as reinforcement leaning, have been put forward to explain choice history biases (Lak et al., 2020). Testing a battery of tasks or designing paradigms that are more closely related to the individuals’ phenomenological experience may prove fruitful. Second, recruitment should address the concurrent issues of heterogeneous, multidimensional profiles and a high degree of correlated/comorbid traits. In the current replication, we validated the AQ with self-reported diagnostic status, yet an alternative strategy would be to collect questionnaire data on potentially correlated traits/diagnoses which may otherwise confound the results. In this case, a transdiagnostic dimensional approach may prove more informative, because various traits (e.g., repetitive behaviors, sensory issues, or intolerance of uncertainty) are shared across subclinical profiles and diagnoses and may be more tightly associated with the mechanisms under investigation.

In conclusion, we show, first, that adaptation to sequential regularities in the environment is underpinned by both an increase in stimulus-independent decision bias and a shift in stimulus-dependent sensitivity that favors predicted information, akin to a perceptual confirmation bias. Second, we find no convincing evidence for reduced choice history bias adaptation in individuals with high AQ scores. Together, our study sheds light on how observers exploit sequential regularities to facilitate perceptual decision-making in structured environments.

## Supplementary Material

Supplement 1

## References

[bib1] Abrahamyan, A., Silva, L. L., Dakin, S. C., Carandini, M., & Gardner, J. L. (2016). Adaptable history biases in human perceptual decisions. *Proceedings of the National Academy of Sciences,* 113(25), E3548–E3557. 10.1073/pnas.1518786113 PMC4922170 27330086

[bib2] Baron-Cohen, S., Wheelwright, S., Skinner, R., Martin, J., & Clubley, E. (2001). The Autism-Spectrum Quotient (AQ): Evidence from Asperger syndrome/high-functioning autism, males and females, scientists and mathematicians. *Journal of Autism and Developmental Disorders,* 31(1), 5–17. 11439754 10.1023/a:1005653411471

[bib3] Barbosa, J., & Compte, A. (2020). Build-up of serial dependence in color working memory. *Scientific reports**,* 10(1), 10959. 32616792 10.1038/s41598-020-67861-2 PMC7331714

[bib4] Bialek, W. (2005). Should you believe that this coin is fair? *arXiv* *preprint* *q-bio/0508044**.*

[bib5] Bogaerts, L., Siegelman, N., Christiansen, M. H., & Frost, R. (2022). Is there such a thing as a “good statistical learner”? *Trends in Cognitive Sciences,* 26(1), 25–37. 34810076 10.1016/j.tics.2021.10.012

[bib6] Bosch, E., Fritsche, M., Utzerath, C., Buitelaar, J. K., & de Lange, F. P. (2022). Adaptation and serial choice bias for low-level visual features are unaltered in autistic adolescents. *Journal of Vision,* 22(6), 1. 10.1167/jov.22.6.1 PMC9078051 35503507

[bib7] Braun, A., Urai, A. E., & Donner, T. H. (2018). Adaptive history biases result from confidence-weighted accumulation of past choices. *Journal of Neuroscience,* 38(10), 2418–2429. 29371318 10.1523/JNEUROSCI.2189-17.2017 PMC5858589

[bib8] Brock, J. (2012). Alternative Bayesian accounts of autistic perception: Comment on Pellicano and Burr. *Trends in Cognitive Sciences,* 16(12), 573–574. 23123383 10.1016/j.tics.2012.10.005

[bib9] Bronfman, Z. Z., Brezis, N., Moran, R., Tsetsos, K., Donner, T., & Usher, M. (2015). Decisions reduce sensitivity to subsequent information. *Proceedings of the Royal Society B: Biological Sciences,* 282(1810), 20150228. 10.1098/rspb.2015.0228 PMC4590468 26108628

[bib10] Brooks, J. L. (2012). Counterbalancing for serial order carryover effects in experimental condition orders. *Psychological Methods,* 17(4), 600–614. 22799624 10.1037/a0029310

[bib11] Cicchini, G. M., Benedetto, A., & Burr, D. C. (2021). Perceptual history propagates down to early levels of sensory analysis. *Current Biology**,* 31(6), 1245–1250. 33373639 10.1016/j.cub.2020.12.004 PMC7987721

[bib12] Cicchini, G. M., Mikellidou, K., & Burr, D. (2017). Serial dependencies act directly on perception. *Journal of Vision**,* 17(14), 6–6. 10.1167/17.14.6 29209696

[bib13] de Lange, F. P., Rahnev, D. A., Donner, T. H., & Lau, H. (2013). Prestimulus oscillatory activity over motor cortex reflects perceptual expectations. *The Journal of Neuroscience,* 33(4), 1400–1410. 23345216 10.1523/JNEUROSCI.1094-12.2013 PMC6618755

[bib14] Feigin, H., Shalom-Sperber, S., Zachor, D. A., & Zaidel, A. (2021). Increased influence of prior choices on perceptual decisions in autism. *ELife,* 10, e61595. 34231468 10.7554/eLife.61595 PMC8289410

[bib15] Fischer, J., & Whitney, D. (2014). Serial dependence in visual perception. *Nature Neuroscience,* 17(5), Article 5. 10.1038/nn.3689 PMC4012025 24686785

[bib16] Fritsche, M., Majumdar, A., Strickland, L., Liebana Garcia, S., Bogacz, R., & Lak, A. (2023). Temporal regularities shape perceptual decisions and striatal dopamine signals. *bioRxiv,* *2023-08**.* 10.1038/s41467-024-51393-8 PMC11330509 39154025

[bib17] Fritsche, M., Mostert, P., & de Lange, F. P. (2017). Opposite effects of recent history on perception and decision. *Current Biology**,* 27(4), 590–595. 28162897 10.1016/j.cub.2017.01.006

[bib18] Fründ, I., Wichmann, F. A., & Macke, J. H. (2014). Quantifying the effect of intertrial dependence on perceptual decisions. *Journal of Vision,* 14(7), 9. 10.1167/14.7.9 24944238

[bib19] Glaze, C. M., Kable, J. W., & Gold, J. I. (2015). Normative evidence accumulation in unpredictable environments. *ELife,* 4, e08825. 26322383 10.7554/eLife.08825 PMC4584511

[bib20] Hermoso-Mendizabal, A., Hyafil, A., Rueda-Orozco, P. E., Jaramillo, S., Robbe, D., & de la Rocha, J. (2020). Response outcomes gate the impact of expectations on perceptual decisions. *Nature Communications,* 11(1), 1057. 10.1038/s41467-020-14824-w PMC7044326 32103009

[bib21] Karvelis, P., Seitz, A. R., Lawrie, S. M., & Seriès, P. (2018). Autistic traits, but not schizotypy, predict increased weighting of sensory information in Bayesian visual integration. *ELife,* 7, e34115. 29757142 10.7554/eLife.34115 PMC5966274

[bib22] Lak, A., Hueske, E., Hirokawa, J., Masset, P., Ott, T., Urai, A. E., & Kepecs, A. (2020). Reinforcement biases subsequent perceptual decisions when confidence is low, a widespread behavioral phenomenon. *ELife,* 9, e49834. 32286227 10.7554/eLife.49834 PMC7213979

[bib23] Lawson, R. P., Rees, G., & Friston, K. J. (2014). An aberrant precision account of autism. *Frontiers in Human Neuroscience,* 8, 302. 24860482 10.3389/fnhum.2014.00302 PMC4030191

[bib24] Lieder, I., Adam, V., Frenkel, O., Jaffe-Dax, S., Sahani, M., & Ahissar, M. (2019). Perceptual bias reveals slow-updating in autism and fast-forgetting in dyslexia. *Nature Neuroscience,* 22(2), 256. 30643299 10.1038/s41593-018-0308-9

[bib25] Lowe, M. X., Stevenson, R. A., Barense, M. D., Cant, J. S., & Ferber, S. (2018). Relating the perception of visual ensemble statistics to individual levels of autistic traits. *Attention, Perception, & Psychophysics,* 80(7), 1667–1674. 10.3758/s13414-018-1580-1 30088256

[bib26] Manassi, M., Murai, Y., & Whitney, D. (2023). Serial dependence in visual perception: A meta-analysis and review. *Journal of Vision**,* 23(8), 18–18. 10.1167/jov.23.8.18 PMC10476445 37642639

[bib27] Martínez-Trujillo, J. C. & Treue, S. (2004). Feature-based attention increases the selectivity of population responses in primate visual cortex. *Current Biology,* 14(9), 744–751. 15120065 10.1016/j.cub.2004.04.028

[bib28] Murai, Y., & Whitney, D. (2021). Serial dependence revealed in history-dependent perceptual templates. *Current Biology,* 31(14), 3185–3191.e3. 34087105 10.1016/j.cub.2021.05.006 PMC8319107

[bib29] Nickerson, R. S. (1998). Confirmation bias: A ubiquitous phenomenon in many guises. *Review of General Psychology,* 2(2), 175–220.

[bib30] Noel, J.-P., Zhang, L.-Q., Stocker, A. A., & Angelaki, D. E. (2021). Individuals with autism spectrum disorder have altered visual encoding capacity. *PLOS Biology,* 19(5), e3001215. 33979326 10.1371/journal.pbio.3001215 PMC8143398

[bib31] Pascucci, D., Mancuso, G., Santandrea, E., Della Libera, C., Plomp, G., & Chelazzi, L. (2019). Laws of concatenated perception: Vision goes for novelty, decisions for perseverance. *PLoS biology**,* 17(3), e3000144. 30835720 10.1371/journal.pbio.3000144 PMC6400421

[bib32] Pascucci, D., Tanrikulu, Ö. D., Ozkirli, A., Houborg, C., Ceylan, G., Zerr, P., & Kristjánsson, Á. (2023). Serial dependence in visual perception: A review. *Journal of Vision**,* 23(1), 9–9. 10.1167/jov.23.1.9 PMC9871508 36648418

[bib33] Pellicano, E., & Burr, D. (2012). When the world becomes ‘too real’: A Bayesian explanation of autistic perception. *Trends in Cognitive Sciences,* 16(10), 504–510. 22959875 10.1016/j.tics.2012.08.009

[bib34] Prat-Ortega, G., & de la Rocha, J. (2018). Selective attention: A plausible mechanism underlying confirmation bias. *Current Biology: CB,* 28(19), R1151–R1154. 30300602 10.1016/j.cub.2018.08.024

[bib36] Press, C., Kok, P., & Yon, D. (2020). The perceptual prediction paradox. *Trends in Cognitive Sciences**,* 24(1), 13–24. 31787500 10.1016/j.tics.2019.11.003

[bib35] Robertson, A. E., & Simmons, D. R. (2013). The relationship between sensory sensitivity and autistic traits in the general population. *Journal of Autism and Developmental Disorders,* 43(4), 775–784. 22832890 10.1007/s10803-012-1608-7

[bib37] Roy, N. A., Bak, J. H., International Brain Laboratory, Akrami, A., Brody, C. D., & Pillow, J. W. (2021). Extracting the dynamics of behavior in sensory decision-making experiments. *Neuron,* 109(4), 597–610.e6. 33412101 10.1016/j.neuron.2020.12.004 PMC7897255

[bib38] Shadlen, M. N., & Newsome, W. T. (2001). Neural basis of a perceptual decision in the parietal cortex (area LIP) of the rhesus monkey. *Journal of Neurophysiology,* 86(4), 1916–1936. 11600651 10.1152/jn.2001.86.4.1916

[bib39] Sinha, P., Kjelgaard, M. M., Gandhi, T. K., Tsourides, K., Cardinaux, A. L., Pantazis, D., & Held, R. M. (2014). Autism as a disorder of prediction. *Proceedings of the National Academy of Sciences,* 111(42), 15220–15225. 10.1073/pnas.1416797111 PMC4210351 25288765

[bib40] Talluri, B. C., Urai, A. E., Tsetsos, K., Usher, M., & Donner, T. H. (2018). Confirmation bias through selective overweighting of choice-consistent evidence. *Current Biology,* 28(19), 3128–3135.e8. 30220502 10.1016/j.cub.2018.07.052

[bib41] Taubert, J., Alais, D., & Burr, D. (2016). Different coding strategies for the perception of stable and changeable facial attributes. *Scientific Reports**,* 6(1), 32239. 27582115 10.1038/srep32239 PMC5007489

[bib42] Urai, A. E., de Gee, J. W., Tsetsos, K., & Donner, T. H. (2019). Choice history biases subsequent evidence accumulation. *ELife,* 8, e46331. 31264959 10.7554/eLife.46331 PMC6606080

[bib43] van Bergen, R. S., & Jehee, J. F. M. (2019). Probabilistic representation in human visual cortex reflects uncertainty in serial decisions. *Journal of Neuroscience,* 39(41), 8164–8176. 31481435 10.1523/JNEUROSCI.3212-18.2019 PMC6786811

[bib44] Van de Cruys, S., Evers, K., van der Hallen, R., van Eylen, L., Boets, B., De-Wit, L., & Wagemans, J. (2014). Precise minds in uncertain worlds: Predictive coding in autism. *Psychological Review,* 121(4), 649–675. 25347312 10.1037/a0037665

[bib45] Wheelwright, S., Auyeung, B., Allison, C., & Baron-Cohen, S. (2010). Defining the broader, medium and narrow autism phenotype among parents using the Autism Spectrum Quotient (AQ). *Molecular Autism,* 1(1), 10. 20678260 10.1186/2040-2392-1-10 PMC2913943

[bib46] Wyart, V., Nobre, A. C., & Summerfield, C. (2012). Dissociable prior influences of signal probability and relevance on visual contrast sensitivity. *Proceedings of the National Academy of Sciences,* 109(9), 3593–3598. 10.1073/pnas.1120118109 PMC3295248 22331901

[bib47] Yu, A. J., & Cohen, J. D. (2008). Sequential effects: Superstition or rational behavior? *Advances in Neural Information Processing Systems,* 21, 1873–1880. 26412953 PMC4580342

