# Supplementary material for: Perceptual confirmation bias and decision bias underlie adaptation to sequential regularities
Source: J Vis. 2024 Feb 21;24(2):5. doi: 10.1167/jov.24.2.5 (PMC10902869; doi:10.1167/jov.24.2.5)
Supplement: Supplement 1 [file jovi-24-2-5_s001.pdf]

Supplementary materials

1. Probabilistic choice model

The amount of regularization (λ = 0.001) was validated through a simulation procedure in study 1, in which the ground-truth history biases were recovered accurately when analyzing responses to repeating stimulus sequences (see Supplementary Figure 1). Specifically, for each participant we estimated psychometric parameters (intercept, slope, lapse rates, and history biases) in the uncorrelated, neutral environment. Based on these, we simulated 100 sets of responses to the stimulus sequences of the repeating environment per participant and estimated the lasso regression on these simulated responses.

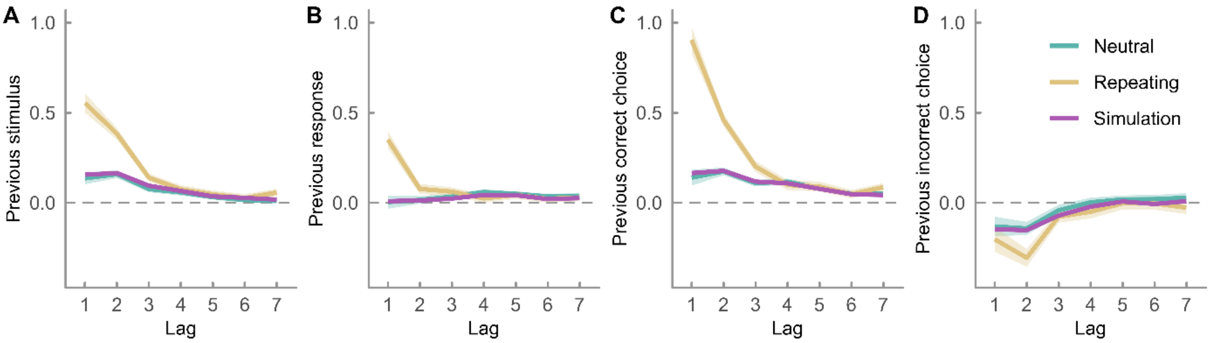

Supplementary Figure 1: Determination of the amount of regularization: simulated responses in the neutral environment result in history biases near zero (**A** previous stimulus, **B** previous response, **C** previous correct choice and **D** previous incorrect choice). Regression weights estimated based on the empirical data in the neutral and repeating environment in study 1 are plotted alongside these for comparison. Shaded areas depict the standard error of the mean.

2. Similar choice history biases for high- and low-AQ participants

The previous correct choice weight consists of previous stimulus and previous response weights, which follow the same trends yet are likewise not significantly correlated with either AQ (previous stimulus adaptation: $\rho_s = -0.12$, p = 0.20; previous response adaptation: $\rho_s = -0.11$, p = 0.22) or GSQ (previous stimulus adaptation: $\rho_s = -0.09$, p = 0.33; previous response adaptation: $\rho_s = -0.02$, p = 0.80; see Supplementary Figure 2) in study 1. The difference in history bias adaptation between the NAP and the neurotypical group in study 1 is primarily attributable to a reduced adaptation of the previous stimulus weight in the NAP group (t(42.85) = -2.7, p=0.01), although the shift in previous response weight tends to be lower as well (t(21.72) = -1.9, p = 0.07; see Supplementary Figure 2). Neither of these differences are significant in the replication sample (previous stimulus adaptation: t(68.38) = 0.08, p = 0.94, previous response adaptation: t(63.672) = -0.41, p = 0.68).

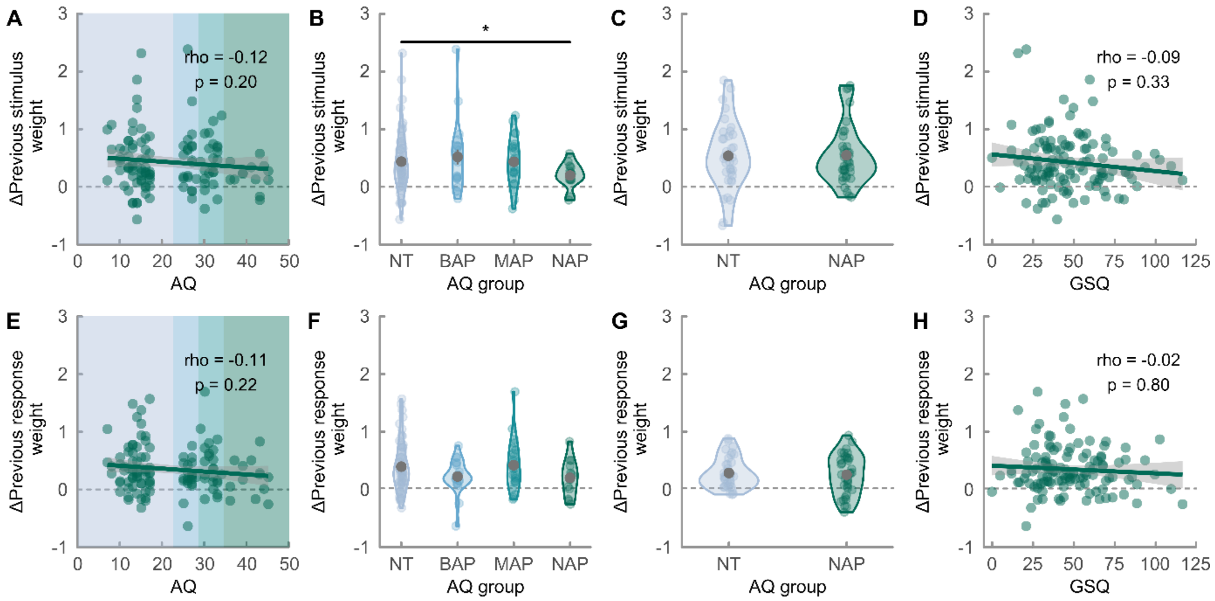

Supplementary Figure 2: Adaptation in one-back previous stimulus and previous response weights for the continuous distribution of AQ scores in study 1 (**A** and **E**, respectively), a categorical classification based on the AQ as neurotypical (NT; AQ<23, n=63), broader autism phenotype (BAP; 22<AQ<29, n=21), medium autism phenotype AQ (MAP; 28<AQ<35, n=23), and narrow autism phenotype (NAP; AQ>34, n=13) in study 1 (**B** and **F**, respectively), participants with AQ scores corresponding to a neurotypical (AQ<17, n=35), and narrow autism phenotype (AQ>34, n=36) in study 2 (**C** and **G**, respectively), and the continuous distribution of GSQ scores in study 1 (**D** and **H**, respectively). Shades of green in the background in **A** and **E** indicate the categorical classification of AQ scores. Asterisks depict the level of significance (* p <0.05, ** p <0.01, *** p <0.001).

Evaluating both environments separately suggests that any trend of individual differences in study 1 arises in the repeating environment (AQ: previous stimulus: ρ_s = -0.13, p = 0.15; previous response: ρ_s = -0.06, p = 0.54; GSQ: previous stimulus: ρ_s = -0.13, p = 0.15; previous response: ρ_s = -0.08, p = 0.37; see Supplementary Figure 3), as opposed to the neutral environment, particularly in the case of the AQ (AQ: previous stimulus: ρ_s = -0.03, p = 0.77; previous response: ρ_s = -0.01, p = 0.93; GSQ: previous stimulus: ρ_s = -0.13, p = 0.17; previous response: ρ_s = 0.11, p = 0.23; see Supplementary Figure 4). As in the continuous distribution of AQ scores, any differences between the NAP and the neurotypical groups are evident in the repeating environment (previous stimulus: t(34.63) = -2.58, p = 0.01); previous response: t(19.14) = -1.00, p = 0.33; see Supplementary Figure 3), but not in the neutral environment (previous stimulus: t(16.47) = -0.38, p = 0.71, ; previous response: t(17.95) = 0.38, p = 0.71 ; see Supplementary Figure 4). In study 2, we find no significant differences in either the neutral (previous stimulus: t(57.79) = -0.46, p = 0.65; previous response: t(67.58) = -0.50, p = 0.62) or the repeating environment (previous stimulus: t(68.71) = -0.19, p = 0.85; previous response: t(68.60) = -0.73, p = 0.47).

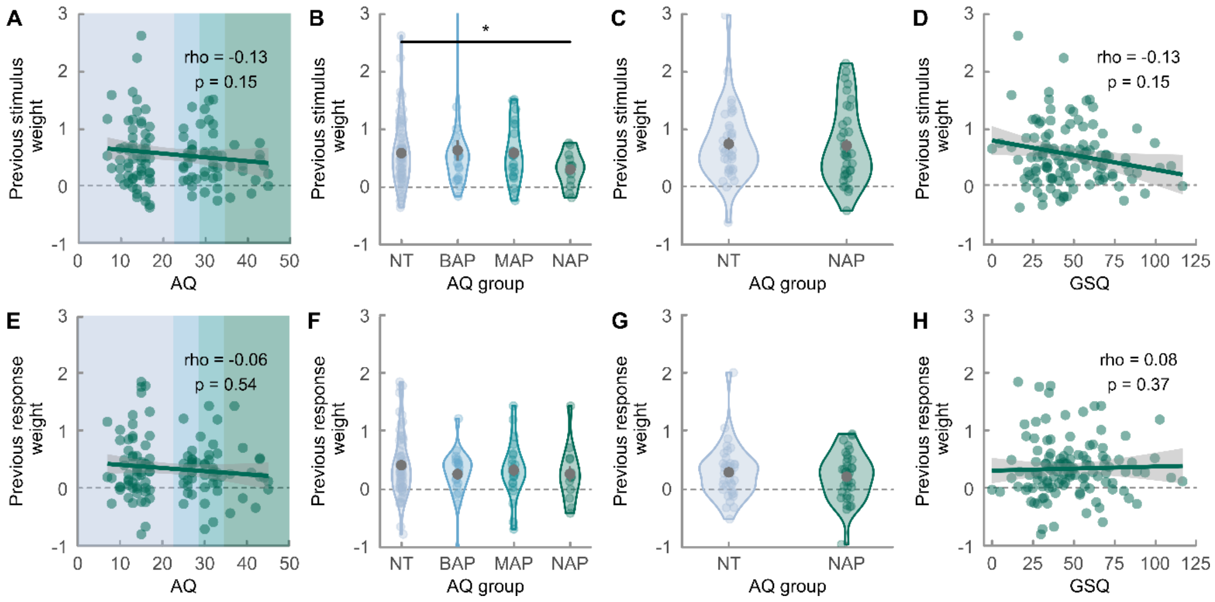

Supplementary Figure 3: One-back previous stimulus and previous response weights in the repeating environment for the continuous distribution of AQ scores in study 1 (**A** and **E**, respectively), a categorical classification based on the AQ as neurotypical (NT; AQ<23, n=63), broader autism phenotype (BAP; 22<AQ<29, n=21), medium autism phenotype AQ (MAP; 28<AQ<35, n=23), and narrow autism phenotype (NAP; AQ>34, n=13) in study 1 (**B** and **F**, respectively), participants with AQ scores corresponding to a neurotypical (AQ<17, n=35), and narrow autism phenotype (AQ>34, n=36) in study 2 (**C** and **G**, respectively), and the continuous distribution of GSQ scores in study 1 (**D** and **H**, respectively). Shades of green in the background in **A** and **E** indicate the categorical classification of AQ scores.

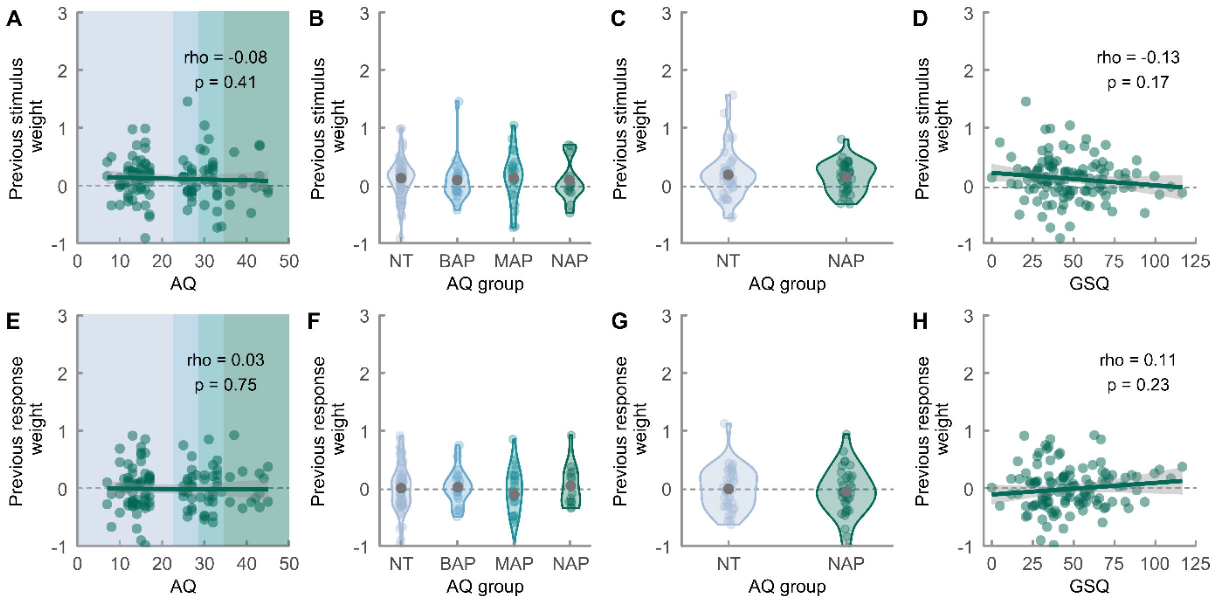

Supplementary Figure 4: One-back previous stimulus and previous response weights in the neutral environment for the continuous distribution of AQ scores in study 1 (**A** and **E**, respectively), a categorical classification based on the AQ as neurotypical (NT; AQ<23, n=63), broader autism phenotype (BAP; 22<AQ<29, n=21), medium autism phenotype AQ (MAP; 28<AQ<35, n=23), and narrow autism phenotype (NAP; AQ>34, n=13) in study 1 (**B** and **F**, respectively), participants with AQ scores corresponding to a neurotypical (AQ<17, n=35), and narrow autism phenotype (AQ>34, n=36) in study 2 (**C** and **G**, respectively), and the continuous distribution of GSQ scores in study 1 (**D** and **H**, respectively). Shades of green in the background in **A** and **E** indicate the categorical classification of AQ scores.

Estimated lapse rates are not associated with autistic-like traits (AQ: ρ_s = -0.00, p = 0.99; GSQ: ρ_s = 0.04, p = 0.68), which holds for categorical differences between the NAP and the neurotypical groups (t(26.39) = -0.82, p = 0.42, study 2: t(61.97) = -0.14, p = 0.89; see Supplementary Figure 5). The current stimulus weights are likewise not significantly correlated with autistic-like traits (AQ: ρ_s = 0.12, p = 0.21; GSQ: ρ_s = 0.14, p = 0.13) or significantly different in the NAP group (t(16.33) = 0.68, p = 0.51, study 2: t(68.14) = 0.34, p = 0.74; see Supplementary Figure 5). The history-independent bias towards clockwise responses tends to be less pronounced in individuals with high levels of autistic-like traits as measured by the AQ (ρ_s = -0.20, p = 0.03), though not the GSQ (ρ_s = 0.08, p = 0.38) in study 1, however this difference was not significant when comparing the NAP and neurotypical groups (t(20.88) = -1.72, p = 0.10, study 2: t(61.87) = -1.17, p = 0.25; see Supplementary Figure 5).

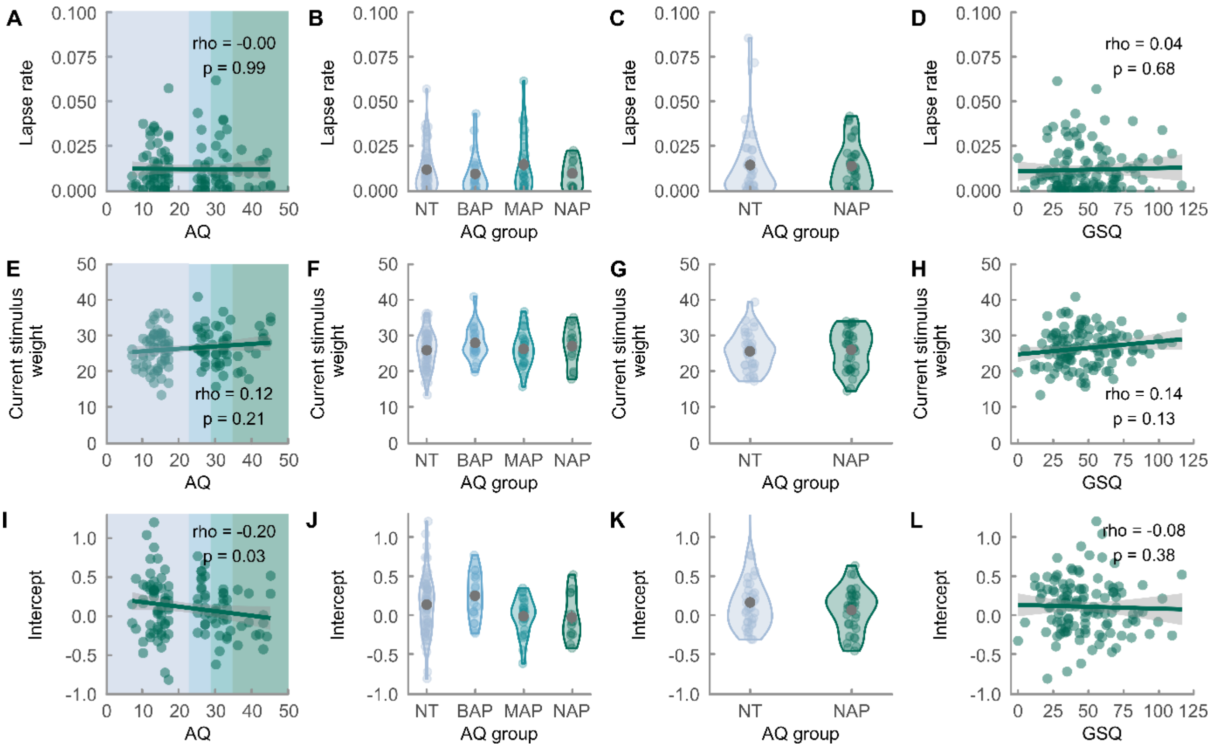

Supplementary Figure 5: Estimated lapse rates, current stimulus and intercept weights in the time-unresolved history bias model averaged across environments for the continuous distribution of AQ scores in study 1 (**A**, **E** and **I**, respectively), a categorical classification based on the AQ as neurotypical (NT; AQ<23, n=63), broader autism phenotype (BAP; 22<AQ<29, n=21), medium autism phenotype AQ (MAP; 28<AQ<35, n=23), and narrow autism phenotype (NAP; AQ>34, n=13) in study 1 (**B**, **F** and **J**, respectively), a categorical classification based on the AQ as neurotypical (AQ<17, n=35), and narrow autism phenotype (AQ>34, n=36) in study 2 (**C**, **G** and **K**, respectively), and the continuous distribution of GSQ scores in study 1 (**D**, **H** and **L**, respectively). Shades of green in the background in **A**, **E**, and **I** indicate the categorical classification of AQ scores.

3. Similar development of choice history biases in high- and low-AQ participants

In study 1, there is a trend for a negative correlation between the AQ and the intercept at the level of the full spectrum ($\rho_s = -0.16$, $p = 0.08$), while there is no such association with the slope ($\rho_s = -0.03$, $p = 0.79$). Directly comparing the NAP and the neurotypical groups reveals a significant effect in the intercept of correct choice weights at the onset of the repeating environment ($t(26.40) = -2.35$, $p = 0.03$), and no significant difference in the slope ($t(14.23) = 0.54$, $p = 0.60$). However, we do not replicate these findings in study 2 (intercept: $t(66.94) = -0.62$, $p = 0.54$; slope: $t(63.64) = -0.15$, $p = 0.88$). The GSQ does not correlate with either of these metrics (intercept: $\rho_s = -0.04$, $p = 0.70$; slope: $\rho_s = -0.03$, $p = 0.78$). It should be noted that the model fit is not uniformly satisfactory in our sample, however this is unlikely to confound the group comparisons, as the R^2 value does not correlate with

autistic-like traits (AQ: ρ_s = -0.008, p = 0.932; NAP vs NT: t(15.33) = -0.76, p = 0.46, study 2: t(60.49) = -1.75, p = 0.09).

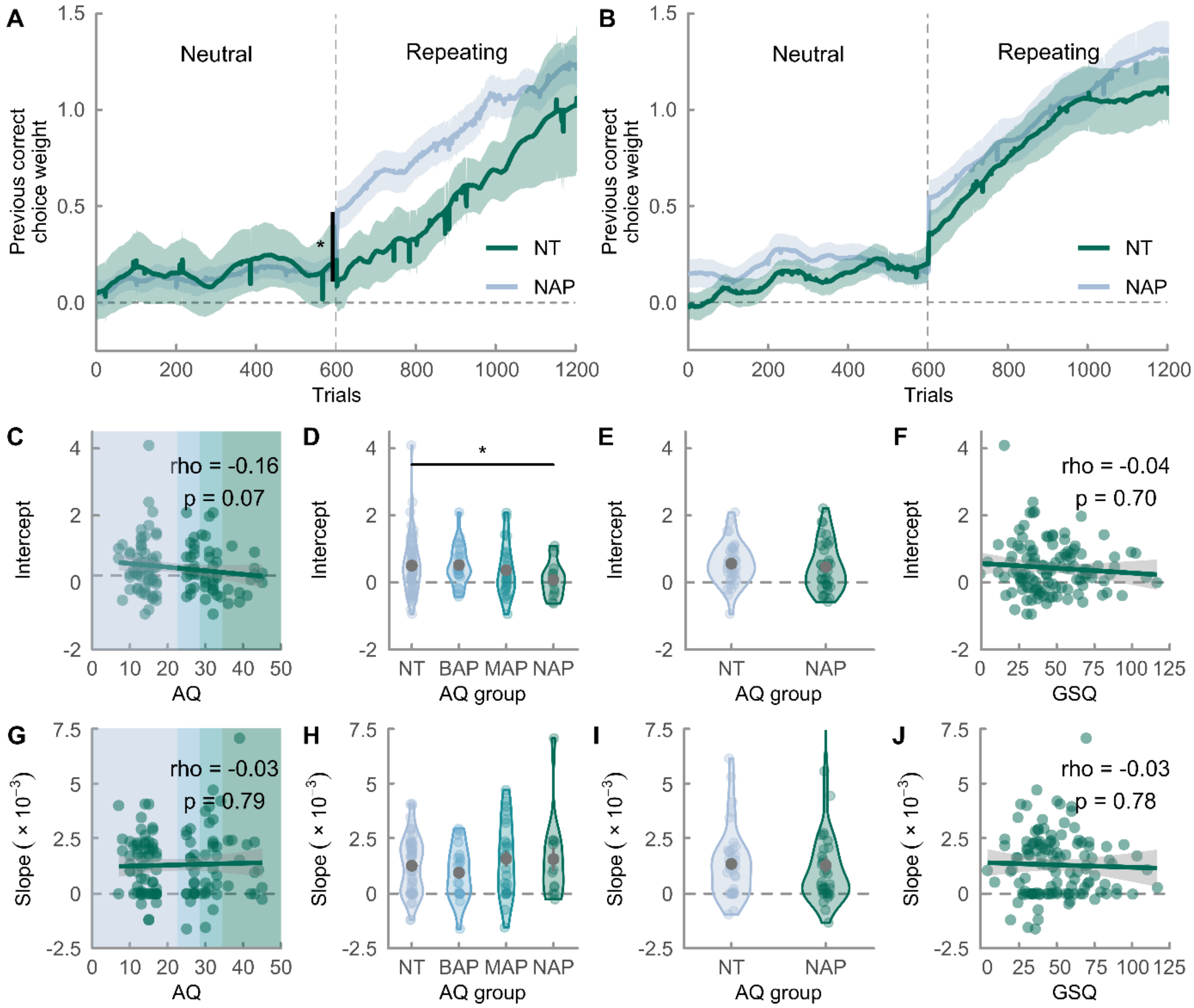

Supplementary Figure 6: Trajectory of the previous correct choice bias across time in an initial neutral and subsequent repeating environment for **A** participants with AQ scores corresponding to a neurotypical (AQ<23, n=63), and narrow autism phenotype (AQ>34, n=13) in study 1 and **B** participants with AQ scores corresponding to a neurotypical (AQ<17, n=35), and narrow autism phenotype (AQ>34, n=36) in study 2. **C-J:** Individual differences in the two indices of learning in the repeating environment, the intercept and the slope of previous correct choice weights, related to autistic-like traits based on the continuous distribution of the AQ in study 1 (**C** and **G**, respectively), a categorical classification based on the AQ as neurotypical (AQ<23, n=63), broader autism phenotype (22<AQ<29, n=21), medium autism phenotype AQ (28<AQ<35, n=23), and narrow autism phenotype (AQ>34, n=13) in study 1(**D** and **H**, respectively), a categorical classification based on the AQ as neurotypical (AQ<17, n=35), and narrow autism phenotype (AQ>34, n=36) in study 2 (**E** and **I**, respectively), and the continuous distribution of GSQ scores in study 1 (**F** and **J**, respectively). Shades of green in the background of **C** and **G** indicate the categorical classification of AQ scores. Error bars depict the standard error of the mean and asterisks the level of significance (* p <0.05, ** p <0.01, *** p <0.001).

4. Similar perceptual confirmation bias and decision bias in high- and low-AQ participants

Finally, we evaluated the individual differences in bias and sensitivity adaptation related to autistic-like traits. In study 1, we find a significant negative correlation at the level of the continuous AQ distribution for stimulus-independent bias adaptation (ρ_s = -0.20, p=0.03), while there is no association with stimulus-dependent sensitivity adaptation (ρ_s = 0.08, p=0.40). Furthermore, a similar result is obtained for the comparison between NAP and NT groups (bias adaptation: t(20.76) = -3.09, p = 0.01; sensitivity adaptation: t(19.71) = -0.19, p = 0.85). However, these findings do not replicate (bias adaptation: t(64.85) = 0.07, p = 0.95; sensitivity adaptation: t(68.71) = 1.33, p = 0.19). There is no significant relationship of the GSQ with bias (ρ_s = -0.01, p=0.95) or sensitivity adaptation (ρ_s = 0.15, p=0.09, r = 0.15).

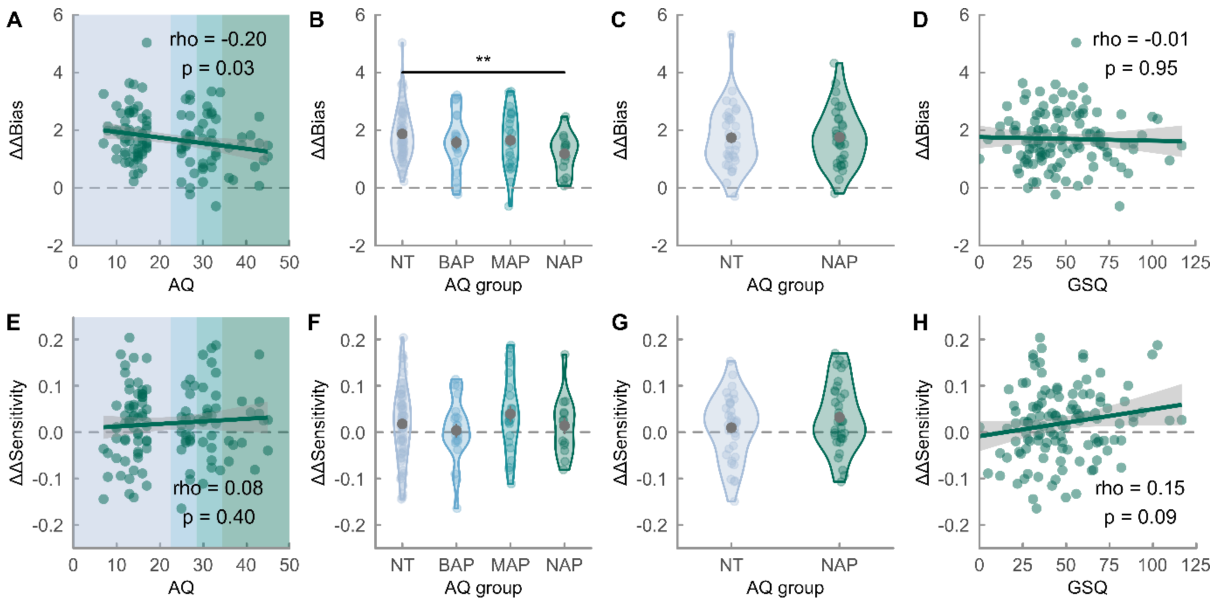

Supplementary Figure 7: Individual differences in adaptation of stimulus-independent bias and stimulus-dependent sensitivity to the sequential regularities in the environment related to autistic-like traits based on the continuous distribution of AQ scores in study 1 (**A** and **E**, respectively), a categorical classification based on the AQ as neurotypical (NT; AQ<23, n=63), broader autism phenotype (BAP; 22<AQ<29, n=21), medium autism phenotype AQ (MAP; 28<AQ<35, n=23), and narrow autism phenotype (NAP; AQ>34, n=13) in study 1 (**B** and **F**, respectively), a categorical classification based on the AQ as neurotypical (AQ<17, n=35), and narrow autism phenotype (AQ>34, n=36) in study 2 (**C** and **G**, respectively), and the continuous distribution of GSQ scores in study 1 (**D** and **H**, respectively). Shades of green in the background **A** and **E** indicate the categorical classification of AQ scores. Error bars depict the standard error of the mean and asterisks the level of significance (* p <0.05, ** p <0.01, *** p <0.001).

5. Control analysis: similar performance in high- and low-AQ

Across both studies, all participants are able to complete the task at a similar level of performance, as mean accuracy and reaction time (RT) are not correlated with the AQ (mean accuracy: $\rho_s = 0.10$, $p = 0.29$; mean RT: $\rho_s = 0.09$, $p = 0.33$), the GSQ (mean accuracy: $\rho_s = 0.09$, $p = 0.34$; mean RT: $\rho_s = 0.05$, $p = 0.61$), or significantly different in the NAP group in either study (mean accuracy study 1: $t(15.05) = -0.03$, $p = 0.98$, study 2: $t(67.83) = 0.60$, $p = 0.55$; mean RT study 1: $t(15.04) = 0.12$, $p = 0.91$, study 2: $t(67.01) = -1.54$, $p = 0.13$; see Supplementary Figure 8).

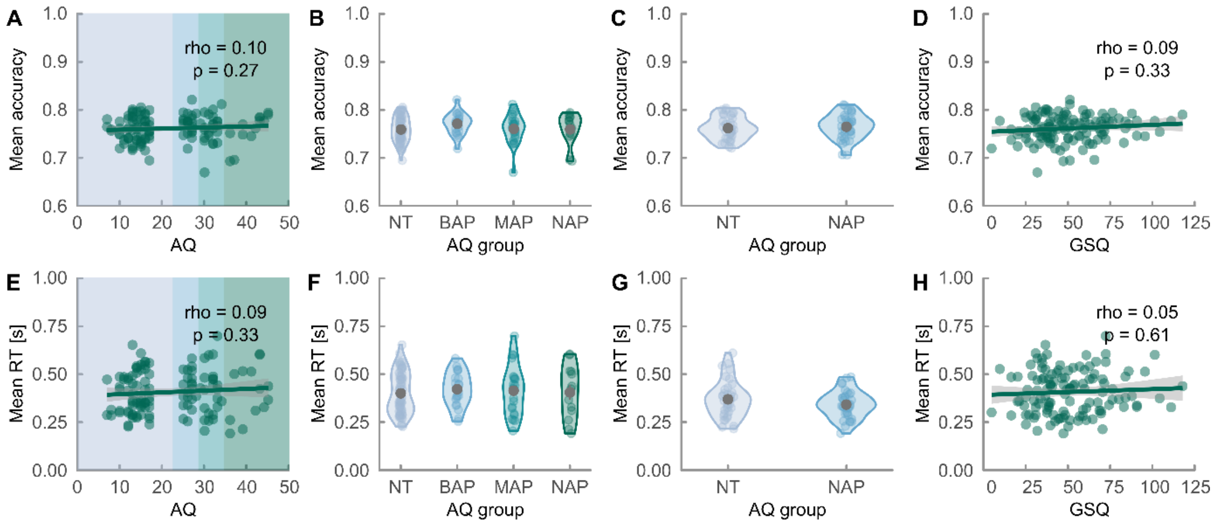

Supplementary Figure 8: Mean accuracy and reaction time averaged across environments for the continuous distribution of AQ scores in study 1 (**A** and **E**, respectively), a categorical classification based on the AQ as neurotypical (NT; AQ<23, n=63), broader autism phenotype (BAP; 22<AQ<29, n=21), medium autism phenotype AQ (MAP; 28<AQ<35, n=23), and narrow autism phenotype (NAP; AQ>34, n=13) in study 1 (**B** and **E**, respectively), a categorical classification based on the AQ as neurotypical (AQ<17, n=35), and narrow autism phenotype (AQ>34, n=36) in study 2 (**C** and **G**, respectively), and the continuous distribution of GSQ scores in study 1. Shades of green in the background in **A** and **E** indicate the categorical classification of AQ scores.
